# Discrimination and Protective Factors to Cognitive Health: Testing NIA’s Health Disparities Framework

**DOI:** 10.1093/geroni/igaa057.2331

**Published:** 2020-12-16

**Authors:** Ernest Gonzales, Cliff Whetung, Jane Lee, Yi Wang

**Affiliations:** 1 New York University, New York, New York, United States; 3 University of Hawai’i, Myron B. Thompson School of Social Work, Honolulu, Hawaii, United States; 4 University of Iowa, Iowa City, Iowa, United States

## Abstract

Cognitive impairment is a worldwide epidemic. Informed by NIA’s Health Disparities Framework, this study investigated interpersonal, behavioral, and sociocultural risk and protective factors associated with cognitive health trajectories. Mixed models examined factors associated with cognitive health with data from the Health and Retirement Study among Whites, Blacks, and Hispanics (2008-2014, N=4,511). A majority of respondents who experienced everyday discrimination attributed it to ageism among this racially and ethnically diverse sample. Stratified mixed models of everyday discrimination by attribution (racism or ageism) revealed worse cognitive functioning. Major lifetime discrimination was not statistically associated with cognitive functioning. Economic factors (education, income, assets) and religious activity protected cognitive functioning and were particularly salient for Blacks and Hispanics. Strategies that bolster individual resilience as well as social policies that address discrimination and structural inequities will likely reduce health disparities and improve population health.

